# 881. Nasal and Plasma SARS-CoV-2 RNA Levels Predict Timing of Symptom Resolution in the ACTIV-2 Trial of Non-hospitalized Adults with COVID-19

**DOI:** 10.1093/ofid/ofac492.074

**Published:** 2022-12-15

**Authors:** Yijia Li, Linda J Harrison, Kara W Chew, Joseph J Eron, Eric S Daar, David A Wohl, Ryan Wu, Carlee Moser, Justin Ritz, Mark Giganti, Arzhang Cyrus Javan, Robert Coombs, Michael D Hughes, Judith S Currier, Davey M Smith, Jonathan Z Li

**Affiliations:** University of Pittsburgh, Pittsburgh, PA; Harvard T.H. Chan School of Public Health, Boston, Massachusetts; David Geffen School of Medicine at University of California, Los Angeles, California; University of North Carolina at Chapel Hill School of Medicine, Chapel Hill, North Carolina; Lundquist Institute at Harbor-UCLA Medical Center, Torrance, California; University of North Carolina at Chapel Hill School of Medicine, Chapel Hill, North Carolina; Harvard T.H. Chan School of Public Health, Boston, Massachusetts; Harvard T.H. Chan School of Public Health, Boston, Massachusetts; Harvard T.H. Chan School of Public Health, Boston, Massachusetts; Harvard T.H. Chan School of Public Health, Boston, Massachusetts; National Institutes of Health, Bethesda, Maryland; University of Washington, Seattle, Washington; Harvard T.H. Chan School of Public Health, Boston, Massachusetts; David Geffen School of Medicine at University of California, Los Angeles, California; University of California, San Diego, La Jolla, California; Brigham and Women's Hospital, Boston, MA

## Abstract

**Background:**

Symptoms during acute COVID-19 can limit daily activities and delay return to work and school. Little is known about the association between SARS-CoV-2 burden in either the upper airway or plasma and the duration of COVID-19 symptoms.

**Methods:**

ACTIV-2/A5401 is a platform trial for COVID-19 treatments in non-hospitalized symptomatic adults enrolled within 10 days of symptom onset. We included participants randomized to placebo from August 2020 to July 2021. Participants self-reported severity of 13 symptoms daily from day 0 (baseline) to 28 as Absent 0, Mild 1, Moderate 2, Severe 3; total symptom score was calculated as the sum of all scores. Anterior nasal (AN) and plasma SARS-CoV-2 RNA levels at day 0 were measured with a quantitative qPCR assay. The relationship between day 0 RNA and time to symptom improvement or resolution (first of 2 consecutive days of all symptoms improved or resolved from day 0, respectively) was evaluated using proportional hazards regression adjusted for time from symptom onset. Time to resolution of distinct symptoms was also assessed.

**Results:**

Among 570 participants randomized to placebo, median age was 48 years, 51% were female, and median time since symptom onset at baseline was 6 days; 7% had prior COVID-19 vaccination. At day 0, AN RNA was detectable in 80% with a median of 4.1 log_10_ copies/ml (n=533, quartiles: 1.7, 6.0) and plasma RNA was detectable in 19% (91/476). Detectable plasma RNA at day 0, but not AN RNA, was associated with more severe symptoms at day 0 (2.4-point higher mean total symptom score, P=0.001). Both high AN (≥6 vs < 2 log_10_ copies/ml, adjusted hazard ratio [aHR] 0.63, P=0.001) and detectable plasma RNA (aHR 0.74, P=0.03) at day 0 predicted delayed symptom improvement. High AN RNA at day 0 also predicted a delay in symptom resolution (aHR 0.59, P=0.001). Both high AN RNA and detectable plasma RNA levels predicted delays in the resolution of cough and shortness of breath. Detectable plasma RNA also predicted delayed body pain resolution.

**Conclusion:**

COVID-19 outpatients with high AN or detectable plasma SARS-CoV-2 RNA at day 0 are more likely to have prolonged symptoms, particularly respiratory symptoms. Additional studies are needed to determine whether the decline in viral load with early treatment impacts symptom duration.

**Disclosures:**

**Kara W. Chew, M.D., M.S.**, Merck Sharp & Dohme: Grant/Research Support|Pardes Bioscences: Advisor/Consultant **Joseph J. Eron, MD**, GSK: Advisor/Consultant|Merck: Advisor/Consultant **Eric S. Daar, M.D.**, Gilead: Advisor/Consultant|Gilead: Grant/Research Support|Merck: Advisor/Consultant|ViiV: Advisor/Consultant|ViiV: Grant/Research Support **David A. Wohl, M.D.**, Gilead: Advisor/Consultant|Gilead: Grant/Research Support|Lilly: Grant/Research Support|ViiV: Advisor/Consultant|ViiV: Grant/Research Support **Judith S. Currier, M.D., MSc**, Merck: Advisor/Consultant **Davey M. Smith, M.D., M.A.S.**, Arena Pharmaceuticals: Advisor/Consultant|Bayer Pharmaceuticals: Advisor/Consultant|Brio Clinical.: Advisor/Consultant|Fluxergy: Advisor/Consultant|Kiadis: Advisor/Consultant|Linear Therapies: Advisor/Consultant|Matrix BioMed: Advisor/Consultant|Model Medicines: Advisor/Consultant|Signant Health: Advisor/Consultant|VxBiosciences: Advisor/Consultant **Jonathan Z. Li, MD, MMSc**, Abbvie: Advisor/Consultant|Merck: Grant/Research Support.

